# Real-world management of ovarian cancer in women living with HIV: treatment exposure, immune monitoring, and care barriers in a five-case series

**DOI:** 10.3389/fonc.2026.1942370

**Published:** 2026-09-30

**Authors:** Yang Zhang, Fuxiang Wang, Mingchuan Guo, Jianyu Wan, Huiyan Liao, Ting Mao, Lu Cui, Wenming Cao, Yutao Gao

**Affiliations:** 1 Shenzhen Third People’s Hospital, Shenzhen, Guangdong, China; 2 South China Hospital, Medical School, Shenzhen University, Shenzhen, Guangdong, China; 3 Shenzhen University General Hospital, Shenzhen, Guangdong, China

**Keywords:** bevacizumab, CD4 + T cells, HIV, ovarian cancer, PARP inhibitor, real-world management, treatment barriers

## Abstract

**Background:**

With improved survival among people living with HIV (PLWH), non-AIDS-defining cancers have become increasingly prominent. Women living with HIV (WLWH) remain underrepresented in ovarian cancer trials, leaving limited evidence on the tolerability of standard therapies, including chemotherapy, anti-VEGF agents, and PARP inhibitors. These patients also face unique care barriers beyond biological uncertainties.

**Methods:**

This retrospective review included five WLWH diagnosed with ovarian cancer (June 2022–March 2024). Three received anticancer treatment; one refused chemotherapy and was lost to follow-up; one had biopsy only. We collected data on demographics, HIV history, tumor characteristics, treatment exposure, adverse events (CTCAE v5.0), CD4^+^/CD8^+^ counts, and HIV RNA, focusing safety and immune analyses on the three treated patients.

**Results:**

Median age was 51 years; four had stage III–IV disease. No treatment-related deaths, persistent HIV rebound, or AIDS-defining events occurred. During first-line paclitaxel/carboplatin, one patient developed grade 3 neutropenia managed with G-CSF, but later developed significant thrombocytopenia on niraparib requiring dose reduction and hospitalization. Among two patients receiving bevacizumab, CD4^+^ patterns diverged: one showed a transient decline (387 to 259 cells/μL) with concurrent nedaplatin; another maintained stable counts (150 to 200 cells/μL). One patient had transient, self-limited HIV RNA elevation concurrent with disease progression, which resolved spontaneously.

**Conclusion:**

This series provides real-world observations that platinum-based and targeted therapies may be delivered to selected WLWH with close monitoring. The heterogeneous CD4^+^ responses highlight the need for individualized immune surveillance rather than attributing changes to any single agent. Integrated oncologic-HIV care and attention to socioeconomic barriers remain critical for this underrepresented population.

## Highlights

- Platinum-based chemotherapy may be tolerated in HIV-infected women with ovarian cancer without HIV progression.
- Niraparib causes manageable thrombocytopenia but does not appear to affect CD4^+^ counts.
- Bevacizumab shows high inter-individual variability on CD4^+^T-cells when combined with chemotherapy.
- Effective viral suppression may be the foundation for safe antitumor therapy in HIV-positive patients.
- Close monitoring of CD4^+^subsets is recommended during bevacizumab plus platinum regimens.

## 1 Introduction

The widespread use of combination antiretroviral therapy has substantially prolonged survival among people living with HIV (1). As survival has improved, non-AIDS-defining cancers have become increasingly important contributors to morbidity and mortality in this population (2, 3). Although ovarian cancer is not classified as an AIDS-defining malignancy, its management in women living with HIV remains insufficiently characterized (4, 5). Evidence is particularly limited because women living with HIV have historically been underrepresented in gynecologic oncology studies and cancer treatment literature (6, 7). As a result, there is little direct evidence regarding the tolerability of standard ovarian cancer treatments, including platinum-containing chemotherapy, bevacizumab, and PARP inhibitors, or their potential effects on HIV viral control and CD4^+^T-cell dynamics in this population (8–10).

Standard treatment for epithelial ovarian cancer commonly includes cytoreductive surgery, platinum-containing chemotherapy, anti-angiogenic therapy such as bevacizumab, and maintenance therapy with PARP inhibitors in selected patients (11–13). Each of these treatment components may raise specific concerns in women living with HIV. Chemotherapy may contribute to myelosuppression and lymphocyte depletion, PARP inhibitors are associated with hematologic toxicity, and anti-VEGF therapy may have immunomodulatory effects (14, 15). These issues are clinically relevant because treatment-related cytopenia or CD4^+^ decline may be difficult to distinguish from HIV progression or antiretroviral treatment failure (16–18).

Previous case reports have suggested that platinum-based chemotherapy can be administered to selected patients with HIV and ovarian cancer without clear deterioration of HIV-related surrogate markers (19). However, these reports were published before the current era of modern ovarian cancer treatment and did not address contemporary combinations involving taxane-platinum chemotherapy, bevacizumab, or PARP inhibitor maintenance.

In addition to biological and treatment-related uncertainties, women living with HIV may face delayed cancer diagnosis, stigma related to HIV status, financial constraints, treatment refusal, and fragmented access to gynecologic oncology and HIV care. These factors may influence treatment uptake, adherence, follow-up, and outcomes. Therefore, real-world case series can provide clinically useful information not only on treatment tolerability but also on immune monitoring and care delivery in this underrepresented population. We report a retrospective five-case series of women living with HIV and ovarian cancer, focusing on treatment exposure, severe adverse events, HIV viral control, CD4^+^ T-cell changes, and real-world barriers to standard care among patients who did and did not receive anticancer treatment.

## 2 Methods

### 2.1 Study design and patients

This retrospective case series is derived from the gynecologic oncology sub-cohort of a larger, ongoing prospective observational study on AIDS-related cancers (“Pathogenesis and Comprehensive Treatment Strategies for AIDS-Related Cancers”; Ethics Approval No. 2026-041-01) conducted at Shenzhen Third People’s Hospital. The parent study is purely observational and does not mandate or randomize any anticancer treatment; all treatment decisions are made by attending physicians based on clinical guidelines and patient-specific factors. This case series specifically reports all five women living with HIV who were diagnosed with ovarian cancer within this sub-cohort between June 2022 and March 2024. Inclusion criteria were: confirmed HIV infection, and ovarian cancer confirmed by histopathology or cytology.

Among the five patients, three received at least one cycle of anticancer treatment, one refused postoperative chemotherapy and was lost to follow-up, and one underwent biopsy only without anticancer treatment. Baseline clinical characteristics were summarized for all five patients, whereas treatment safety, CD4^+^ T-cell dynamics, and HIV viral load changes were described only for the three treated patients. Care pathways and barriers to standard treatment were described for all five patients.

### 2.2 Data collection

All data for this case series were extracted from the parent study database and supplemented by direct chart review of electronic medical records. Demographic characteristics, HIV-related history, antiretroviral therapy regimens, HIV RNA viral load, CD4^+^/CD8^+^ T-cell counts, tumor histology, FIGO stage, CA125 level, genetic testing results, treatment regimens, and clinical outcomes were collected. The data extraction and analysis were performed on fully anonymized datasets. Treatment exposure included surgery, chemotherapy, bevacizumab, PARP inhibitors, and subsequent systemic therapy. Adverse events were graded according to CTCAE v5.0. CD4^+^/CD8^+^ T-cell counts and HIV RNA viral load were collected before and during anticancer treatment when available. HIV RNA viral load was measured by the laboratory using PCR-based assays and was reported in IU/mL according to the laboratory platform. The lower limit of detection ranged from 20 to 40 IU/mL across testing periods. The assay used in our laboratory is calibrated against the WHO International Standard for HIV-1 RNA, and results are reported in IU/mL.

### 2.3 Follow-up and definition of HIV disease progression

Follow-up was defined as the time from ovarian cancer diagnosis to death or last clinical contact. The last follow-up date was April 1, 2026.

HIV disease progression was defined as a confirmed HIV RNA increase of more than 1 log10 from baseline lasting for more than one month, or a CD4^+^ T-cell decline of more than 30% or to below 200 cells/μL lasting for more than three months without recovery, in the absence of an alternative explanation such as chemotherapy-related lymphopenia, intercurrent infection, perioperative stress, or tumor progression.

### 2.4 Ethical considerations

This study is a retrospective case series derived from medical records of patients treated at Shenzhen Third People’s Hospital between June 2022 and March 2024. The research protocol for this retrospective analysis was reviewed and approved by the Ethics Committee of Shenzhen Third People’s Hospital (Approval No. 2026-041-01) on March 17, 2026. All data were extracted from routine clinical records and fully anonymized prior to analysis. Because this study involves no more than minimal risk to subjects, does not affect clinical care, and involves the use of de-identified data from existing medical records, the institutional ethics committee, under the same approval (No. 2026-041-01), granted a waiver of individual informed consent for the retrospective chart review, in accordance with Chinese regulations (Guidelines for Ethical Review of Biomedical Research Involving Human Subjects, 2023, Article 32) and the Declaration of Helsinki.

For the three treated patients whose detailed clinical courses are presented in this report, written informed consent for the publication of de-identified clinical information, laboratory data, treatment timelines, and imaging was obtained from the patients or their legal representatives. The study was conducted in accordance with the Declaration of Helsinki, the Chinese Guidelines for Ethical Review of Biomedical Research Involving Human Subjects (2016, 2023), and institutional policies. The study protocol and waiver of consent were approved by the institutional ethics committee. The ethics approval letter is provided in the **Supplementary Material** (**Supplementary Figures S2**–**S4**).

## 3 Results

### 3.1 Baseline characteristics of patients

The median age of the five patients was 51 years. Four patients had FIGO stage III–IV disease, and one had FIGO stage IIB disease. The median duration from HIV diagnosis to ovarian cancer diagnosis was 4 years. Four patients were receiving antiretroviral therapy at the time of ovarian cancer diagnosis, including three receiving combination antiretroviral therapy and one receiving lamivudine monotherapy, which was considered non-standard treatment. Case 3 was already on this non-standard regimen upon transfer to our center from another hospital, and her ART was optimized by our infectious disease team during her cancer treatment course. Histologically, three patients had high-grade serous carcinoma, one had low-grade serous carcinoma, and one had endometrioid carcinoma with concomitant endometrial carcinoma. Case 1 had FIGO stage IIIC high-grade serous carcinoma. Case 3 underwent primary cytoreductive surgery in January 2023. The initial diagnosis was high-grade serous carcinoma, but a second surgery for a metastatic lesion revealed carcinosarcoma (**Supplementary Figures S5**–**S7**). Because Case 4 refused postoperative chemotherapy and was lost to follow-up, and Case 5 underwent biopsy only without anticancer treatment, subsequent treatment safety and immune-dynamic analyses focused on Cases 1-3. However, Cases 4 and 5 were retained in the overall description because they illustrate real-world barriers to standard ovarian cancer care in women living with HIV. Baseline characteristics are shown in **Table 1**.

**Table 1:** Baseline characteristics of five patients with HIV and ovarian cancer.

Case	Age, years	HIV duration, years	ART regimen at diagnosis	Baseline CD4^+^, cells/μL	Tumor histology	FIGO stage	CA125, U/mL	Genetic testing	Anticancer treatment
1	51	0.5	TDF + 3TC + EFV; later switched to bictegravir-based ART	407	High-grade serous carcinoma	IIIC	2438	BRCA wild type; HRD not done	Yes
2	45	15	Prior NVP + TDF + 3TC; switched to Triumeq 1 year before diagnosis	668	Endometrioid adenocarcinoma with endometrial carcinoma	IIB	229	BRCA wild type; HRD negative	Yes
3	65	0.2	3TC monotherapy, non-standard regimen	150	High-grade serous carcinoma initially; carcinosarcoma at second surgery	IIIC	785.3	HRD negative, score 11.98; germline testing not done	Yes
4	36	10	TDF + 3TC + NVP	776	Low-grade serous carcinoma	IIIA	3065	Not done	No; lost to follow-up
5	56	20	TDF + 3TC + EFV	430	High-grade serous carcinoma	IV	124	Not done	No; biopsy only

ART, antiretroviral therapy; TDF, tenofovir disoproxil fumarate; 3TC, lamivudine; EFV, efavirenz; NVP, nevirapine; HRD, homologous recombination deficiency.

### 3.2 Treatment exposure, adverse events, and clinical outcomes

Among the three treated patients, all received platinum-containing chemotherapy. Case 1 received paclitaxel plus carboplatin as first-line chemotherapy and later received niraparib maintenance therapy and bevacizumab-containing treatment at recurrence. Case 2 received liposomal doxorubicin plus carboplatin because of pre-existing neuropathy and later received nab-paclitaxel, nedaplatin, and sintilimab after disease progression. Case 3 received nab-paclitaxel plus carboplatin and bevacizumab-containing treatment.

No treatment-related death or severe opportunistic infection was observed among the three treated patients. During first-line paclitaxel plus carboplatin, Case 1 developed grade 3 neutropenia, with an absolute neutrophil count nadir of 0.72 × 10^9^/L, which was managed with G-CSF and supportive care without treatment delay. The platelet nadir during first-line chemotherapy was 106 × 10^9^/L and therefore was not classified as grade ≥3 thrombocytopenia. Later, during niraparib exposure, Case 1 developed clinically significant thrombocytopenia, with a platelet nadir of 26 × 10^9^/L, requiring dose interruption and hospitalization. After recovery, niraparib was resumed at 100 mg every other day and subsequently escalated to 100 mg daily, which was tolerated with stable platelet counts.

Case 2 experienced rapid disease progression and died three months after diagnosis. Case 2 had a transient, self-limited HIV RNA elevation to 1.51 × 10³ IU/mL concurrent with radiographic progression, which subsequently resolved without ART modification. Case 3 did not develop grade 3 or higher toxicity during bevacizumab-containing treatment and had stable primary lesions at last follow-up.

Treatment exposure, severe adverse events, and outcomes among the three treated patients are summarized in **Table 2**. The treatment timelines and major clinical events are illustrated in **Figure 1**.

**Table 2:** Anticancer treatment, severe adverse events, and outcomes among treated patients.

Case	Surgery	First-line systemic therapy	Bevacizumab exposure	PARP inhibitor exposure	Subsequent therapy	Grade ≥3 adverse events	Clinical outcome
1	Suboptimal debulking, R2, September 2022	Paclitaxel + carboplatin, 8 cycles	Yes; at recurrence, combined with nedaplatin	Niraparib 200 mg daily, later reduced because of thrombocytopenia	Nab-paclitaxel + nedaplatin + bevacizumab; followed by bevacizumab maintenance	Grade 3 neutropenia during first-line chemotherapy; clinically significant thrombocytopenia during niraparib exposure	Partial response; ongoing maintenance therapy
2	Optimal debulking, R0, April 2023	Liposomal doxorubicin + carboplatin, 2 cycles	No; not used because of hemoptysis	No	Nab-paclitaxel + nedaplatin + sintilimab	No confirmed grade ≥3 treatment-related adverse event recorded; hemoptysis precluded bevacizumab use	Death due to rapid tumor progression
3	Suboptimal debulking, January 2023	Nab-paclitaxel + carboplatin, 6 cycles	Yes; from cycle 2 for 6 cycles, followed by 4 cycles of maintenance	No	Gemcitabine + docetaxel	None	Stable primary lesions; carcinosarcoma diagnosed after second surgery

Case 1 received eight cycles of first-line chemotherapy because of high tumor burden and sustained response. During first-line chemotherapy, Case 1 required G-CSF support for grade 3 neutropenia but completed all eight cycles without delay. Case 2 completed only two cycles of first-line chemotherapy before radiographic progression, consistent with primary platinum-refractory disease. The transient HIV RNA elevation in Case 2 subsequently resolved without ART modification and did not meet the predefined criterion for HIV disease progression.

**Figure 1:**
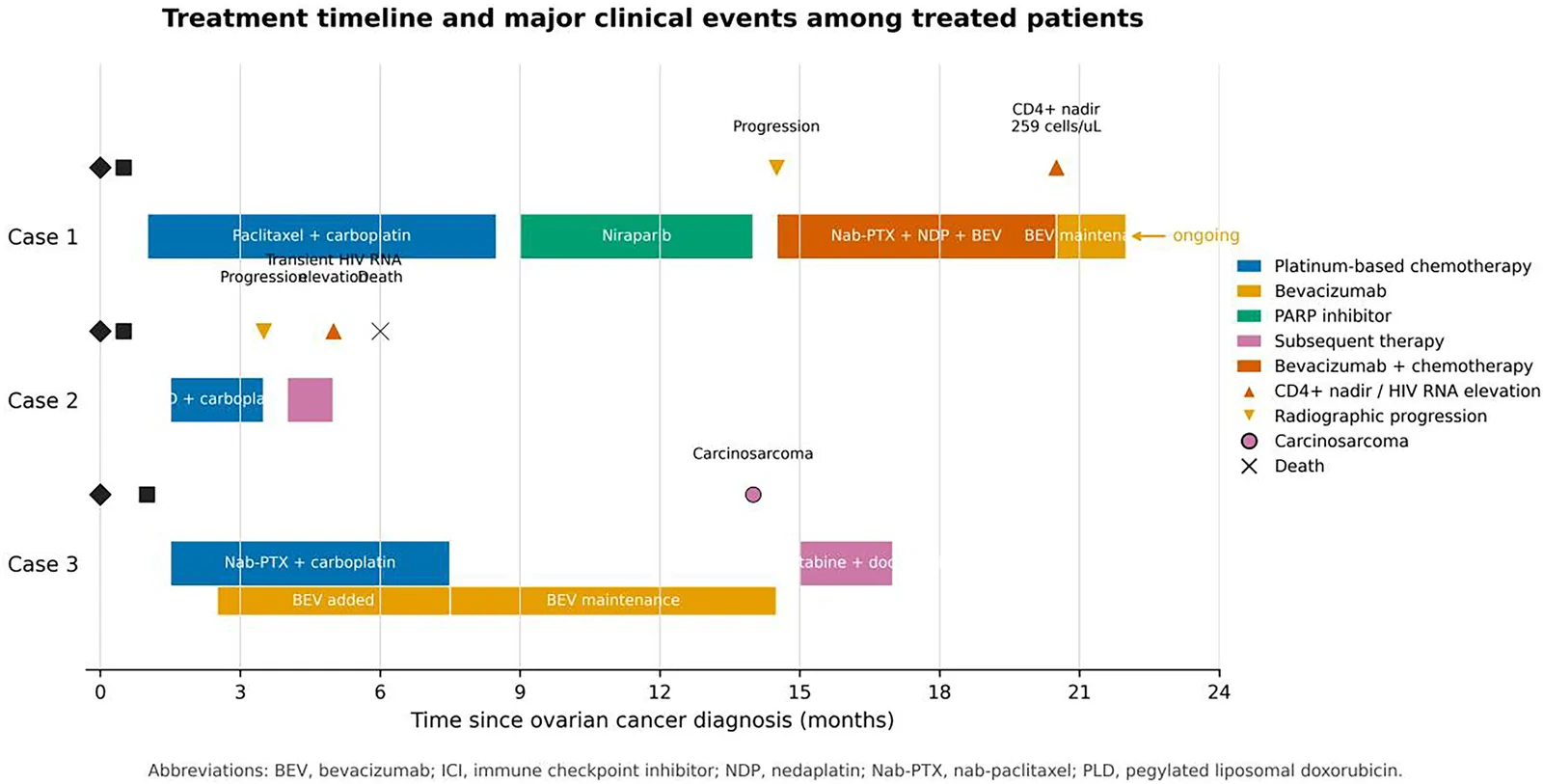
Treatment timeline and major clinical events among the three treated patients. Horizontal bars represent treatment durations for each patient. Blue bars indicate platinum-containing chemotherapy; orange bars indicate bevacizumab exposure; green bar indicates PARP inhibitor maintenance; purple bars indicate subsequent therapy; red bar indicates chemotherapy combined with bevacizumab at recurrence. Triangles indicate CD4^+^ nadir or transient HIV RNA elevation; inverted triangles indicate radiographic disease progression; circles indicate pathological diagnosis of carcinosarcoma at second surgery; crosses indicate death. Case 1 remained on bevacizumab maintenance at last follow-up.

### 3.3 CD4^+^ T-cell dynamics and HIV viral load

None of the three treated patients developed persistent HIV viral rebound or an AIDS-defining event during anticancer treatment. CD4^+^ T-cell changes differed between patients. In Case 1, the CD4^+^ count declined from 387 to 259 cells/μL, corresponding to a 33.1% decrease, during bevacizumab plus nedaplatin treatment. The CD4^+^ count subsequently increased after discontinuation of this regimen. Because bevacizumab was administered concurrently with nedaplatin, this transient CD4^+^ decline cannot be attributed to bevacizumab alone. In Case 3, the baseline CD4^+^ count was low, approximately 150 cells/μL, but remained relatively stable during bevacizumab-containing treatment. Despite the low baseline CD4^+^ count, no persistent HIV viral rebound or AIDS-defining event occurred. Case 1 also received niraparib, during which CD4^+^ counts remained similar to baseline despite hematologic toxicity. These observations suggest that CD4^+^ T-cell monitoring may be clinically useful during anticancer treatment in women living with HIV, but the small number of treated patients precludes causal inference. Refer to **Table 3**.

**Table 3:** CD4^+^ T-cell and HIV RNA dynamics during anticancer treatment.

Case	Baseline CD4^+^, cells/μL	CD4^+^ during chemotherapy, cells/μL	CD4^+^ before bevacizumab, cells/μL	CD4^+^ nadir during or after bevacizumab, cells/μL	Last recorded CD4^+^, cells/μL	HIV RNA status during treatment	AIDS-defining event
1	407	468 after 4 cycles	387	259, 33.1% decrease	339, recovered after treatment interruption	Persistently undetectable	No
2	668	>500, exact value not recorded	Not applicable	Not applicable	Not available	Transient elevation to 1.51 × 10³ IU/mL, followed by suppression	No
3	150	175	180	165, no marked decline	190	Persistently undetectable	No

In Case 1, the CD4^+^ decline lasted approximately six weeks and did not meet the predefined criterion for HIV disease progression. The transient HIV RNA elevation in Case 2 occurred concurrently with radiographic progression and subsequently resolved without ART modification. Niraparib exposure in Case 1 was not accompanied by an apparent CD4^+^ decline. Case 3 maintained stable CD4^+^ counts during bevacizumab-containing treatment despite a low baseline CD4^+^ count.

CD4^+^ T-cell changes according to treatment phase are illustrated in **Figure 2**. The figure shows a transient CD4^+^ decline in Case 1 during bevacizumab plus nedaplatin, relatively stable CD4^+^ counts in Case 3 during bevacizumab-containing treatment despite a low baseline CD4^+^ count, and incomplete but stable available CD4^+^ data in Case 2.

**Figure 2:**
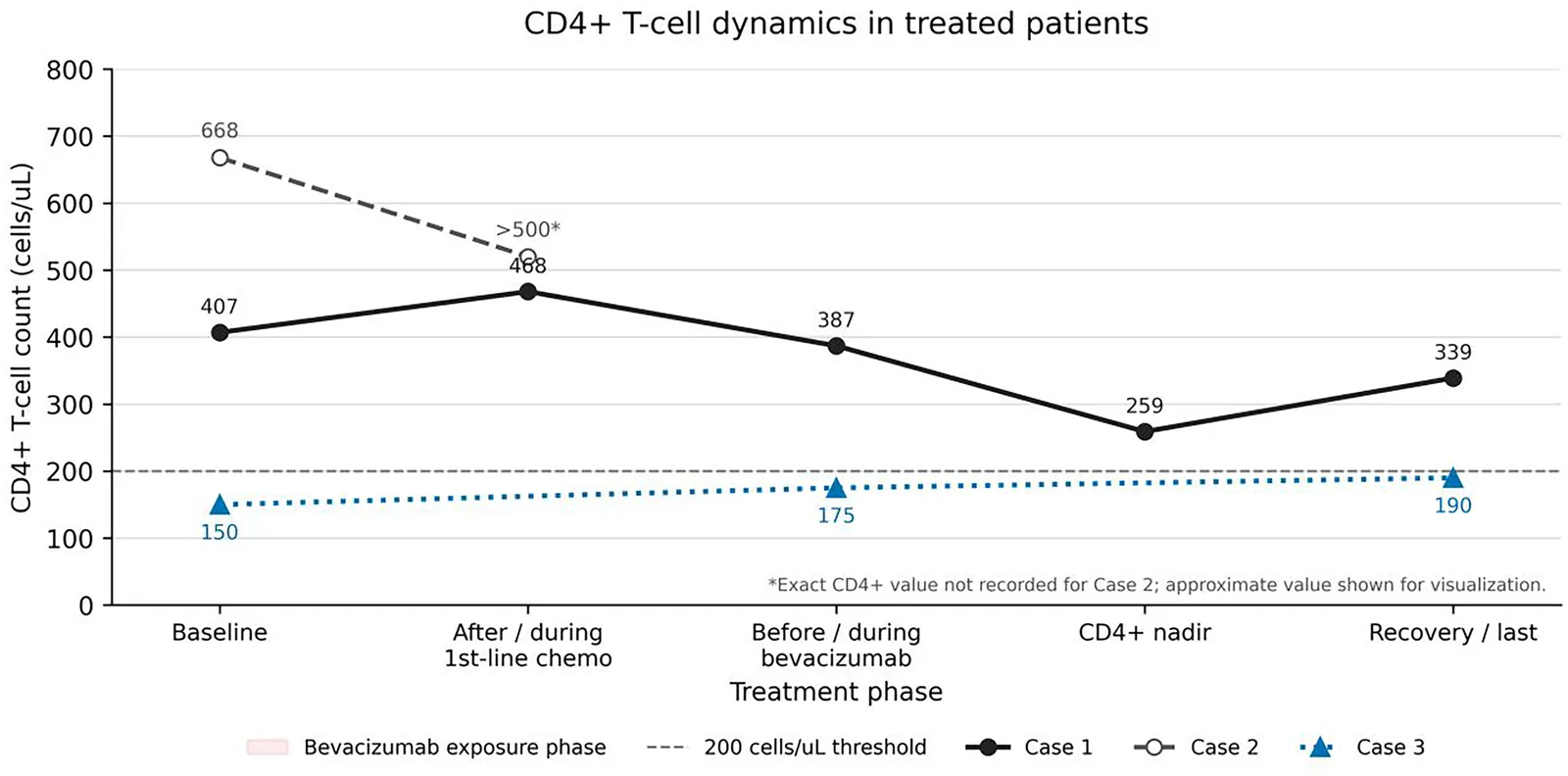
CD4^+^ T-cell dynamics in the three treated patients. CD4^+^ counts are plotted according to treatment phase rather than calendar time to facilitate comparison across patients. Case 1 showed a transient CD4^+^ decline from 387 to 259 cells/μL during bevacizumab plus nedaplatin, followed by recovery after treatment interruption or modification. Case 3 maintained relatively stable CD4^+^ counts during bevacizumab-containing treatment despite a low baseline CD4^+^ count. Case 2 had incomplete CD4^+^ data during treatment; only baseline and approximate values during first-line chemotherapy were recorded. The grey dashed line indicates the clinical threshold of 200 cells/μL. The pink shaded area indicates the period of bevacizumab exposure.

### 3.4 Real-world care barriers in untreated or incompletely treated patients

Two of the five patients did not receive standard systemic anticancer treatment. Case 4 underwent surgery for FIGO stage IIIA low-grade serous carcinoma but refused postoperative chemotherapy and was subsequently lost to follow-up. Case 5 had biopsy-confirmed advanced high-grade serous carcinoma but did not receive anticancer treatment because of economic constraints and remained alive with tumor at last contact. In Case 2, economic considerations also influenced treatment choices after rapid disease progression. These cases illustrate that real-world outcomes in women living with HIV and ovarian cancer may be shaped not only by tumor biology and immune status but also by treatment access, financial burden, follow-up continuity, and patient preference. Care pathways and major barriers among all five patients are summarized in **Table 4**.

**Table 4:** Real-world care pathways and barriers among five women living with HIV and ovarian cancer.

Case	Standard treatment recommended	Treatment received	Main barrier	Follow-up status	Clinical implication
1	Surgery, platinum-containing chemotherapy, and maintenance therapy	Multimodal treatment including chemotherapy, niraparib, and bevacizumab	Hematologic toxicity during treatment	Ongoing follow-up	Multimodal therapy was delivered with close monitoring
2	Surgery and systemic therapy for recurrent or progressive disease	Surgery and limited systemic therapy	Rapid tumor progression and economic constraints	Died of tumor progression	Tumor biology and treatment access affected outcome
3	Surgery, platinum-containing chemotherapy, and bevacizumab	Multimodal treatment including chemotherapy and bevacizumab	Low baseline CD4^+^ count; non-standard ART requiring infectious disease monitoring	Ongoing follow-up	HIV suppression and immune monitoring were clinically important
4	Postoperative systemic therapy and structured follow-up	Surgery only	Treatment refusal and economic concern	Lost to follow-up	Loss to follow-up limited disease monitoring
5	Systemic anticancer treatment	Biopsy only	Economic constraints	Alive with tumor at last contact	Access barriers prevented standard care

## 4 Discussion

### 4.1 Principal findings

In this retrospective five-case series, three women living with HIV received anticancer treatment for ovarian cancer. No persistent HIV viral rebound, AIDS-defining event, treatment-related death, or severe opportunistic infection was observed among the treated patients. All three treated patients received platinum-containing chemotherapy, and no persistent HIV viral rebound or AIDS-defining event was observed, although hematologic toxicity occurred in one patient. CD4^+^ T-cell patterns varied during targeted therapy exposure: one patient had a transient CD4^+^ decline during bevacizumab plus nedaplatin, whereas another maintained stable CD4^+^ counts during bevacizumab-containing treatment. One patient received niraparib and developed thrombocytopenia, but CD4^+^ counts remained stable during exposure.

In addition to treatment and immune-monitoring findings, this series highlights real-world barriers to standard care, including economic constraints, treatment refusal, and loss to follow-up. These factors are clinically important because they may shape outcomes in women living with HIV and ovarian cancer as much as biological or treatment-related factors.

These findings should be interpreted cautiously because the study included only five patients, and the safety and immune-dynamic analyses were limited to three treated patients. The observations are therefore descriptive and hypothesis-generating rather than confirmatory.

### 4.2 Platinum-containing chemotherapy in women living with HIV

Previous reports have suggested that platinum-based chemotherapy can be administered to selected patients with HIV and ovarian cancer without clear worsening of HIV-related surrogate markers. In the present series, all three treated patients received platinum-containing chemotherapy, and no persistent HIV viral rebound or AIDS-defining event was observed. These findings suggest that standard ovarian cancer chemotherapy may be delivered to selected patients with controlled HIV infection under careful monitoring, but they do not establish the general safety of platinum-containing chemotherapy in this population.

Case 1 developed grade 3 neutropenia during first-line paclitaxel/carboplatin despite a baseline CD4^+^ count of 407 cells/μL, which was managed with G-CSF support without treatment delay. This observation suggests that hematologic toxicity from chemotherapy itself requires close monitoring in this population, even before the introduction of targeted agents. However, completion of treatment without delay in this case suggests that standard-dose chemotherapy may be delivered with appropriate supportive care in selected patients.

Case 2 progressed rapidly after two cycles of liposomal doxorubicin plus carboplatin and died of tumor progression. This outcome most likely reflects aggressive platinum-refractory ovarian cancer rather than HIV progression. However, because the sample size was extremely small, treatment efficacy could not be evaluated. The transient HIV RNA elevation observed in Case 2, which subsequently resolved without ART modification, may reflect a stress-related or tumor-burden-related phenomenon rather than true virologic failure. This observation underscores the importance of repeating HIV RNA testing before making ART changes in patients with advanced cancer.

### 4.3 PARP inhibitor exposure

Case 1 received niraparib maintenance therapy after first-line platinum-containing chemotherapy and developed clinically significant thrombocytopenia, with a platelet nadir of 26 × 10^9^/L. The thrombocytopenia improved after treatment interruption, hospitalization, supportive management, and dose reduction. After platelet recovery, niraparib was resumed at 100 mg every other day and was later gradually re-escalated to 100 mg daily, which was ultimately tolerated with stable platelet counts.

Importantly, CD4^+^ T-cell counts remained stable during niraparib exposure despite the occurrence of thrombocytopenia. This observation suggests that niraparib-related hematologic toxicity can be managed with standard monitoring and individualized dose adjustment in selected women living with HIV. However, because this finding was based on a single patient, it cannot exclude potential immune effects of PARP inhibitors in other patients living with HIV.

The stable CD4^+^ counts during niraparib exposure are reassuring, but no conclusion can be drawn regarding lineage-specific toxicity from a single case. Given the known hematologic toxicity profile of niraparib, complete blood counts, especially platelet counts, should be closely monitored when PARP inhibitors are used in women living with HIV (20). This may be particularly important in patients with prior chemotherapy exposure, limited marrow reserve, low baseline blood counts, or concomitant antiretroviral drugs with potential hematologic toxicity (21).

### 4.4 CD4^+^ T-cell changes during bevacizumab-containing therapy

Two patients in this series received bevacizumab-containing treatment and showed different CD4^+^ T-cell patterns. Case 1 experienced a transient CD4^+^ decline of 33.1% during bevacizumab plus nedaplatin, followed by recovery after treatment interruption or modification. In contrast, Case 3 had a low baseline CD4^+^ count but maintained relatively stable CD4^+^ values during bevacizumab-containing treatment.

The CD4^+^ decline observed in Case 1 should not be attributed to bevacizumab alone, because nedaplatin was administered concurrently and chemotherapy-related lymphocyte toxicity could have contributed. Other factors, including tumor burden, prior treatment exposure, marrow reserve, intercurrent illness, perioperative stress, and immune redistribution, may also have affected peripheral CD4^+^ T-cell counts (22). Importantly, the contrasting CD4^+^ trajectory in Case 3, who received bevacizumab with carboplatin without a marked CD4^+^ decline, further argues against a simple direct bevacizumab-induced lymphopenia. Whether different platinum agents or treatment backbones have distinct effects on lymphocyte dynamics remains uncertain and cannot be determined from this case series.

Although Case 3 had a low baseline CD4^+^ count, stable HIV viral suppression and absence of AIDS-defining events suggest that bevacizumab-containing treatment may be considered in selected patients under close oncologic and HIV monitoring. However, this observation is based on a single case and should not be generalized.

VEGF blockade may influence immune regulation through effects on dendritic cells, regulatory T cells, endothelial activation, and lymphocyte trafficking (23–25). However, the clinical significance of these mechanisms in women living with HIV receiving ovarian cancer treatment remains uncertain. Therefore, the present observations should be regarded as a signal supporting immune monitoring rather than evidence of a direct bevacizumab-induced CD4^+^ decline.

### 4.5 HIV control and clinical monitoring

None of the treated patients experienced persistent HIV viral rebound or AIDS-defining events during anticancer treatment. This observation suggests that effective viral suppression may be important when delivering ovarian cancer treatment to women living with HIV. In this series, one patient had a transient HIV RNA elevation during rapid tumor progression, but the elevation subsequently resolved without ART modification and did not meet the predefined criterion for HIV disease progression. This finding highlights the importance of confirming HIV RNA changes and interpreting them in the context of tumor progression, systemic illness, treatment stress, and adherence before diagnosing virological failure (26, 27).

Case 3 was receiving lamivudine monotherapy, which is not a standard antiretroviral regimen. The sustained viral suppression observed in this patient while on a non-standard regimen should not be interpreted as support for antiretroviral monotherapy. The case was transferred to our center on this regimen, and it underscores the critical need for early involvement of HIV specialists to optimize ART before and during anticancer therapy (28).

In patients receiving chemotherapy, PARP inhibitors, or bevacizumab, HIV RNA, CD4^+^ T-cell counts, CD8^+^ T-cell counts, CD4/CD8 ratio, and complete blood counts should be monitored regularly (29, 30). Abrupt CD4^+^ decline should prompt evaluation for chemotherapy-related lymphopenia, intercurrent infection, medication adherence, HIV viral rebound, and other reversible causes before modifying antiretroviral therapy or interrupting anticancer treatment. This coordinated approach may reduce unnecessary ART changes and help distinguish treatment-related immune fluctuation from true HIV disease progression.

### 4.6 Public health, pathological, and real-world care implications

Beyond treatment tolerability, this case series highlights several real-world barriers in the care of women living with HIV and ovarian cancer. Two of the five patients did not receive standard postoperative or systemic anticancer treatment because of economic constraints, treatment refusal, or loss to follow-up. These barriers are clinically important because delayed treatment, incomplete treatment, and fragmented follow-up may have a greater impact on outcomes than HIV infection itself in some patients.

The transition of the pathological diagnosis in Case 3 from high-grade serous carcinoma to carcinosarcoma is a clinically important finding, as carcinosarcoma is known to be more aggressive and is associated with a poorer prognosis. As shown in **Supplementary Figures S5**–**S7**, the initial ovarian biopsy demonstrated pure high-grade serous carcinoma with diffuse p53 overexpression (**Supplementary Figure S5**), while the second surgery specimen revealed a biphasic tumor with both epithelial carcinomatous and mesenchymal chondrosarcomatous components—confirming the diagnosis of carcinosarcoma (**Supplementary Figures S6**, **S7**).This evolution underscores the importance of considering tumor heterogeneity and repeat biopsy in cases of rapid progression.

Women living with HIV may face overlapping vulnerabilities, including cancer-related symptom burden, concerns about treatment toxicity, financial pressure, stigma related to HIV status, and limited access to integrated gynecologic oncology and infectious disease care (31, 32). In this context, CD4^+^ T-cell decline or transient HIV RNA elevation during cancer treatment may generate anxiety for patients and clinicians and may be misinterpreted as HIV progression or antiretroviral treatment failure (33). Repeated HIV RNA testing, longitudinal immune monitoring, and communication between oncologists and HIV specialists are therefore essential before changing antiretroviral therapy or interrupting anticancer treatment (34).

These observations support a multidisciplinary model of care for women living with HIV and gynecologic malignancies. Such a model should include early gynecologic cancer evaluation, optimization of antiretroviral therapy, proactive management of hematologic toxicity, psychosocial support, financial counseling when available, and structured follow-up. Although this study cannot determine population-level outcomes, it underscores the need for greater inclusion of women living with HIV in gynecologic oncology research and for real-world evidence addressing both clinical and social determinants of cancer care. A proposed multidisciplinary care framework derived from these observations is provided in **Supplementary Figure S1**.

### 4.7 Limitations

This study has several limitations. It was a single-center retrospective case series with only five patients, and only three received anticancer treatment. The sample size was too small to evaluate treatment efficacy or establish the safety of specific regimens. The effects of bevacizumab could not be separated from those of concurrent chemotherapy, particularly in Case 1. CD4^+^ testing intervals were not uniform, ART regimens were heterogeneous, and there was no HIV-negative control group. Two patients did not contribute to the treatment safety analysis because one refused postoperative chemotherapy and one underwent biopsy only. Beyond clinical factors, socioeconomic barriers substantially influenced outcomes in this series, with two patients declining or forgoing standard therapy. This reflects the real-world challenges in managing this vulnerable population and underscores the need for integrated supportive care and efforts to reduce treatment disparities.

Because this study was descriptive and hypothesis-generating, no causal relationship can be inferred between bevacizumab, PARP inhibitor exposure, platinum chemotherapy, and changes in CD4^+^ T-cell counts. The heterogeneity of treatment exposure and follow-up reflects real-world clinical practice, in which treatment decisions are shaped not only by tumor biology and immune status but also by patient preference, economic factors, and access to multidisciplinary care.

Despite these limitations, this series provides preliminary clinical observations regarding ovarian cancer treatment in women living with HIV, a population that remains underrepresented in prospective ovarian cancer trials.

## 5 Conclusion

In this real-world five-case series, three women living with HIV received platinum-containing chemotherapy for ovarian cancer. No persistent HIV viral rebound or AIDS-defining event was observed during treatment, although hematologic toxicity occurred in one patient. CD4^+^ T-cell counts remained stable during niraparib exposure in one patient, while a transient CD4^+^ decline was observed in another patient during bevacizumab plus nedaplatin. Because of concurrent chemotherapy and the contrasting CD4^+^ stability observed in another patient receiving bevacizumab with carboplatin, this CD4^+^ decline cannot be attributed to bevacizumab alone.

These findings should be interpreted as preliminary and hypothesis-generating. Close monitoring of HIV RNA, CD4^+^ T-cell counts, and hematologic toxicity is warranted when standard ovarian cancer therapies are used in women living with HIV. The series also underscores the importance of integrated gynecologic oncology and HIV care, with attention to treatment access, adherence, financial barriers, and structured follow-up. Larger multicenter studies are needed to clarify treatment tolerability, immune effects, and clinical outcomes in this rare and underrepresented population.

## Data Availability

The original contributions presented in the study are included in the article/**Supplementary Material**. Further inquiries can be directed to the corresponding authors.
