# Supplementary material for: Real-world management of ovarian cancer in women living with HIV: treatment exposure, immune monitoring, and care barriers in a five-case series
Source: Front Oncol. 2026 Sep 30;16:1942370. doi: 10.3389/fonc.2026.1942370 (PMC13661414; doi:10.3389/fonc.2026.1942370)
Supplement: Supplementary file 1 [file DataSheet1.docx]

**Supplementary Material**

***Supplementary Figure S1.*** *Proposed multidisciplinary care framework for women living with HIV and ovarian cancer.*

**Supplementary Note 1: Relationship to Parent Study and Ethical Framework**

This case series (Cases 1–5) is a retrospective sub-analysis of five patients from the parent study "Pathogenesis and Comprehensive Treatment Strategies for AIDS-Related Cancers" (Ethics Approval No. 2026-041-01). The parent study is a multicenter, prospective observational cohort study approved by the Ethics Committee of Shenzhen Third People's Hospital on March 17, 2026.

The parent study protocol (Version 1.0, dated March 16, 2026) explicitly states that it is observational and does not mandate any study-driven interventions. All treatment decisions are made by attending physicians based on clinical guidelines and patient-specific factors. The research team is only responsible for observing, recording, and analyzing the patient's natural disease course.

All data for this case series were extracted from the parent study database and fully anonymized prior to analysis. The retrospective analysis of anonymized data was conducted in compliance with institutional and national regulations. Specific written informed consent for the publication of de-identified clinical information was obtained from the three treated patients.

**Supplementary Note 2**

***Supplementary Figure S2.*** *Ethics approval letter from the Ethics Committee of Shenzhen Third People's Hospital (Approval No. 2026-041-01) - page 1 of 3.*

***Supplementary Figure S3.*** *Ethics approval letter -page 2 of 3.*

***Supplementary Figure S4.*** *Ethics approval letter -page 3 of 3.*

**Supplementary Figure S5. Initial ovarian biopsy showing high-grade serous carcinoma.**

卵巢高级别浆液性癌_10.00X

Figure S5 A High-grade serous carcinoma with papillary and solid growth patterns

卵巢高级别浆液性癌_10.00X-2

Figure S5 A (alternative) High-grade serous carcinoma (alternative view)

卵巢高级别浆液性癌_20.00X

Figure S5 B High-grade serous carcinoma showing marked nuclear atypia

卵巢高级别浆液性癌_20.00X-2

Figure S5 B (alternative) High-grade serous carcinoma (alternative view)

卵巢高级别浆液性癌_20.00X-3

Figure S5 B (alternative) High-grade serous carcinoma (alternative view)

高级别浆液性癌p53_10.00X

Figure S5 C p53 immunohistochemistry showing diffuse strong nuclear staining

高级别浆液性癌 p53 20.00X

Figure S5 D p53 immunohistochemistry (higher magnification)

**Supplementary Figure S6. Second surgery specimen revealing carcinosarcoma with heterologous mesenchymal differentiation.**

骨外+浆液性癌_10.00X

Figure S6 A Biphasic morphology with both carcinomatous and sarcomatous components

高级别浆液性癌_10.00X-3

Figure S6 B Carcinomatous component (epithelial element)

骨外软骨肉瘤_20.00X

Figure S6 C Chondrosarcomatous component with atypical chondrocytes

**Supplementary Figure S7. Detailed histopathological features of the sarcomatous component in carcinosarcoma.**

高级别浆液性癌_10.00X-2

Figure S7 A Carcinomatous component with glandular and solid architecture

高级别浆液性癌_20.00X

Figure S7 B	Sarcomatous component showing cartilaginous differentiation
